# The development of bioactive peptides from dietary proteins as a dipeptidyl peptidase IV inhibitor for the management of type 2 diabetes

**DOI:** 10.7603/s40681-015-0014-9

**Published:** 2015-08-13

**Authors:** Chia-Ling Jao, Chuan-Chuan Hung, Yu-Shan Tung, Pei-Yi Lin, Meng-Chun Chen, Kuo-Chiang Hsu

**Affiliations:** 1 Department of Food and Beverage Management, Tung-Fang Design University, No. 110, Tung-Fang Road, 829 Kaohsiung, Taiwan; 2 Department of Nutrition, China Medical University, 404 Taichung, Taiwan; 3 Department of Health and Nutrition Biotechnology, Asia University, 413 Taichung, Taiwan

**Keywords:** Antidiabetic effect, DPP-IV inhibitory peptide, *In silico*, Type 2 diabetes mellitus

## Abstract

One of the new approaches to the management of type 2 diabetes mellitus (T2DM) consists of orally administered dipeptidyl peptidase-IV (DPP-IV) inhibitors. These synthetic drug inhibitors are reported to have some side effects and that subsequently limits their applications. There is a growing interest to develop natural DPP-IV inhibitors that will be potent without undesirable side effects. Many *in vitro* and some *in vivo* studies have highlighted the potential of food-derived peptides functioning as effective DPPIV inhibitors. Bioactive peptides within original food-derived proteins are inactive but can be activated by being released during food processing (by enzymatic hydrolysis or fermentation) or during gastrointestinal digestion. Hence, the utilization of computer-aided techniques as screening tools may be helpful in predicting the potential of food proteins as precursors of DPP-IV inhibitory peptides. This paper reviews the current literature on DPP-IV inhibitory peptides, focusing on their *in vitro* activity and *in vivo* antidiabetic effects. In addition, the feasibility of various *in silico* approaches is also summarized in this review.

## 1. Introduction

Type 2 diabetes mellitus (T2DM) is the most prevalent metabolic disorder that is characterized by insulin insensitivity as a result of impaired insulin secretion, insulin resistance, and eventual pancreatic beta-cell failure [1, 2]. T2DM leads to an increase in blood glucose levels [3]. It is reported that 387 million people are living with diabetes mellitus (DM), and, furthermore, DM caused 4.9 million deaths in 2014. It is estimated that by 2035 the number of people affected by DM will reach 592 million with over 90% of them being T2DM [4]. T2DM is a complex disease and is a leading cause of cardiovascular disease, blindness, kidney failure, and lower limb amputation [5]. Therefore, it is important to develop effective strategies to manage T2DM in preventing further progression of this disease and its associated complications.

One of the novel strategies for the treatment of T2DM consists of orally administered dipeptidyl peptidase-IV (DPP-IV; EC 3.4.14.5) inhibitors. The enzyme DPP-IV, a serine protease, has a specificity to remove dipeptides from the N-terminus of substrate poly-peptides by cleaving postproline or alanine residues [6]. It is present in a variety of tissues, particularly epithelial tissues of the liver, kidney and small intestine, and exists as a soluble circulating form [7]. This multifunctional enzyme is implicated in several biological processes, including the degradation of chemokines, neuropeptides, and incretin hormones, e.g. glucose-dependent insulinotropic polypeptide (GIP) and glucagon-like peptide-1 (GLP-1) [8]. Both the incretin hormones have the potential to stimulate insulin secretion from the islet beta-cell in a glucose- dependent manner [9]. GLP-1 also has some other physiological actions, such as stimulation of insulin biosynthesis, inhibition of glucagon secretion, decrease of gastric emptying and food intake, and enhancement of satiety [10-12]. In normal humans, the incretin effect is mediated mainly by GIP and GLP-1 and is estimated to be responsible for 50-70% of the insulin response to the oral administration of glucose [13, 14]. In T2DM patients, the incretin effect is impaired or absent because of both reductions in the secretion of GLP-1 and pancreatic responses to GIP [13, 15]. In addition, the two incretin hormones have short half-lives of only 1-2 min following their secretion in response to the ingestion of nutrients because of the degradation by the action of DPP-IV [16] that results in the loss of their insulinotropic activity [17]. Therefore, the use of DPP-IV inhibitors is a novel approach for the management of T2DM because in using DPP-IV inhibitors the insulinotropic response to GLP-1 is still preserved in patients [18].

A number of DPP-IV inhibitors have been described, many of which have been designed based upon the substrate specificity of the enzyme, potency, oral bioavailability, and duration of action [19]. DPP-IV inhibitors are classified as peptidomimetics andnon-peptidomimetics. Valine pyrrolidide and isoleucine thiazolidide are the initial peptidomimetic DPP-IV inhibitors that mimic the N- terminal dipeptide as the cleaving site of the enzyme. Vildagliptin, saxagliptin, sitagliptin, and alogliptin are approved to be antidi- abetic agents by the United States and Europe. Although most synthetic DPP-IV inhibitors are generally well-tolerated, some side effects have been recently reported, including nasopharyn- gitis, headaches, and urinary infections [20, 21]. Therefore, it is important for T2DM therapy to develop a potent DPP-IV inhibitor from natural sources without adverse effects. Dietary proteins, the precursors of a variety of bioactive peptides, have been recognized to improve various aspects of human health [22, 23]. The bioactive peptides embedded within the sequence of a protein can be released by enzymatic hydrolysis, microbial fermentation, and processing methods. A wide range of short-length peptides from dietary protein, e.g. milk [24, 25], rice [26], amaranth, wheat, soybean [27], and fish byproducts [28, 29], have been reported to possess *in vitro* DPP-IV inhibitory activity. Research on some DPP-IV inhibitory peptides has shown that they are effective at stimulating insulin secretion and improving glycemic control in animal models and subjects with T2DM [30-32]. These effective peptides, having lengths that vary from 3-15 amino acids, particularly involved the presence of at least one proline within the sequence and mostly in the penultimate N-terminal residue [33, 34]. According to the findings in the literature, therefore, developing a tool to assist in the selection of food proteins embedded with DPP-IV inhibitory peptides previously identified is important as well as efficient in predicting the potential of these proteins to manage T2DM.

In recent years, computational *(in silico)* methods have been demonstrated to be useful in predicting the potential of proteins as precursors of peptides in various bioactivities, such as DPP-IV and angiotensin-I converting enzyme (ACE) inhibitory activities [35-38]. There are two major *in silico* approaches: the frequency of the occurrence of bioactive peptides within a dietary protein [37], and binding modes by docking analysis [27]. The former is calculated as the number of previously identified bioactive peptides that are found in a given dietary protein; furthermore, the simulation of protein hydrolysis by a bioinformatics tool, e.g. BIOPEP database and program, to find peptides that can be released by a given enzyme is efficient to classify proteins as potential sources of bioactive fragments [36]. The latter, the ligand- enzyme and molecular docking analysis, can simulate the binding and interactions between peptides and enzymes such as DPP-IV and ACE in order to evaluate the inhibitory effects of the peptides [27]. The findings from these two *in silico* analyses may provide the basis to exploit food proteins as naturally occurring materials for the generation of peptides with DPP-IV inhibitory activity [37]. In the present review, the role and potential of bioactive peptides derived from food proteins to be DPP-IV inhibitors are considered. Future perspectives also receiveattention in this review.

## 2. In vitro DPP-IV inhibitory activity of peptides from dietary proteins

Proteins are well known as precursors of a range of bioactive peptides. The bioactive peptides that are derived from food proteins show a physiological effect in the body in addition to their nutritional values. The fact that proteins are precursors of bioactive peptides is particularly attractive for the development of functional foods because bioactive peptides are commonly used food ingredients and are of natural origins. Food protein-derived peptides can be used as potent alternative pharmaceuticals to chemosynthetic drugs due to an ever-increasing interest in safety and economical usage. The bioactive peptides embedded in their parent proteins are in inactive forms and are activated once released from the proteins by enzymatic or acidic hydrolysis, and their biological activity is determined by their native amino acid composition and sequence [39].

Many DPP-IV inhibitory peptides have been discovered in the enzymatic hydrolysates of various food proteins, including milk proteins [31, 40, 41], rice bran [26], amaranth proteins [27], ham [42], and fish proteins [28, 29]. Table 1 shows a summary of *in vitro* DPP-IV inhibitory peptides that are ordered by increasing IC_50_ value as reported in the literature.

Diprotin A, the most potent DPP-IV inhibitory peptide found to date, was isolated from culture filtrates of *Bacillus cereus* BMF673-RF1 [43]. Diprotin A was produced by reciprocally shaking a culture of the strain BMF673-RF1 for 2 days in a medium containing 1% glucose, 1% glycerol, 1% potato starch, 0.5% polypepton, 0.5% meat extract, 0.5% NaCl, 0.32% CaC〇_3_, and 0.05% silicon oil KM-70. Diprotin A was identified Ile-Pro-Ile and had an IC_50_ value of 3.5 pM. A whey protein concentration rich in ß-lactoglobulin hydrolyzed by trypsin was fractionated by semi-preparative RP-HPLC [45]. Two (F2 and F3) of the six obtained fractions showed greater DPP-IV inhibitory activities with IC50 values of 367.3 and 86.0 pg/mL, respectively. A peptide, Ile-Pro-Ala-Val-Phe, in fraction F3 was identified as having an IC_50_ value of 44.7 pM. The peptide Ile-Pro-Ala was also obtained from ß-lactoglobulin hydrolysates using proteinase K [41]. This peptide showed one amino acid change in its tis sequence as compared to diprotin A Ile-Pro-Ile (position 3). However, the substitution at position 3 resulted in a weakening of the inhibitory effect versus diprotin A (IC_50_ value 49 pM versus 3.5 pM). Interestingly, the two peptides, Ile-Pro-Ala-Val-Phe and Ile-Pro-Ala, were both derived from ß-lactoglobulin and showed similar DPP- IV inhibitory activity probably due to the same sequences in the first three amino acid residues [41, 45]. A water-soluble extract of a gouda-type cheese ripened for 12 months and then was separated by RP-HPLC [31]. Forty-six peptide sequences contained in the DPP-IV inhibitory fractions from the extract were identified, and two of these peptides were synthesized and showed greater DPP-IV inhibitory activity. Leu-Pro-Gln-Asn-Ile-Pro-Pro-Leu and Leu-Pro-Gln were both derived from ß-casein, and their IC_50_ values against DPP-IV were 46 and 82 pM, respectively. The two peptides also had the same sequences in the first three amino acid residues, but their IC_50_ values were quite different. Tuna cooking juice has been used for the generation of DPP-IV inhibitory peptides [28]. Three peptides, Pro-Gly-Val-Gly-Gly-Pro-Leu-Gly-Pro-Ile- Gly-Pro-Cys-Tyr-Glu (1412.7 Da), Cys-Ala-Tyr-Gln-Trp-Gln- Arg-Pro-Val-Asp-Arg-Ile-Arg (1690.8 Da), and Pro-Ala-Cys- Gly-Gly-Phe-Try-Ile-Ser-Gly-Arg-Pro-Gly (1304.6 Da), were identified to show potent DPP-IV inhibitory activities, and their IC50 values ranged from 78 to116 pM. These peptides have longer lengths than typical DPP-IV inhibitory peptides. The results demonstrate that the DPP-IV inhibitory activity of peptides is determined by the composition and sequence of amino acids rather than their length.

## 3. In vivo antidiabetic effect of peptides

To date, only a small number of studies have been done on the *in vivo* antidiabetic effects of peptides from dietary proteins. These studies are listed in Table *2* *.* The trypsin-treated β-lactoglobulin was used to evaluate its hypoglycemic efficacy in the C57BL/6 mice model [40]. Mice received the control (0.01 M Tris-HCl buffer), trypsin-treated ß-lactoglobulin (300 mg/kg), or sitagliptin phosphate hydrate (3 mg/kg; positive control) by oral administration 1 h prior to an oral glucose tolerance test. The ß-lactoglobulin hydrolysate and sitagliptin significantly decreased the blood glucose level at 30 min over the 2-h post-prandial period *(P* < 0.01), and they both also significantly lowered AUC_120 min_ values as compared to the control (P < 0.01). The ß-lactoglobulin hydrolysate showed an *in vitro* IC_5(I_ value of 210 ^M against DPP-IV; and a hexapeptide (Val-Ala-Gly-Thr-Trp-Tyr) isolated from the hydrolysate showed an IC50 value of 174 pM. However, the IC50 value against DPP-IV of sitagliptin phosphate hydrate was 19.6 nM and extremely stronger than the hexapeptide. A peptide, Leu-Pro- Gln-Asn-Ile-Pro-Pro-Leu, obtained from a water-soluble extract of gouda-type cheese had an *in vitro* value of 46 pM against DPP-IV and was used for further evaluation *in vivo* in rats [31]. The peptide, orally administered to rats at a dose of 300 mg/kg, significantly lowered peripheral plasma glucose concentrations at 30 and 60 min after glucose loading as compared to the control group *(P* < 0.01). However, the plasma insulin level at each sampling point during the 2-h post-prandial period did not differ significantly between the two groups.

**Table 1:** 一 Protein-derived DPP-IV inhibitory peptides ordered by increasing IC_50_ value.

Peptide sequence	IC_50_ (μM)	Reference
Ile-Pro-Ile	3.5	[43]
Ile-Pro-Ile-Gln-Tyr	35.2	[38]
Trp-Arg	37.8	[44]
Trp-Lys	40.6	[44]
Gly-Pro-Ala-Gly	41.1	[29]
Gly-Pro-Gly-Ala	41.9	[29]
Trp-Leu	43.6	[44]
Trp-Pro	44.5	[44]
Ile-Pro-Ala-Val-Phe	44.7	[45]
Leu-Pro-Gln-Asn-Ile-Pro-Pro-Leu	46	[31]
Ile-Pro-Ala	49	[41]
Cys-Ala-Tyr-Gln-Trp-Gln-Arg-Pro-Val-Asp-Arg-Ile-Arg	78	[28]
Leu-Pro-Gln	82	[31]
Pro-Ala-Cys-Gly-Gly-Phe-Tyr-Ile-Ser-Gly-Arg-Pro-Gly	96.4	[28]
Pro-Gly-Val-Gly-Gly-Pro-Leu-Gly-Pro-Ile-Gly-Pro-Cys-Tyr-Glu	116	[28]
His-Leu	143	[46]
Leu-Pro-Gln-Asn-Ile-Pro-Pro	160	[31]
Val-Ala	168	[46]
Phe-Pro-Gly-Pro-Ile-Pro-Asp	260	[31]
Phe-Leu	399	[46]
Ile-Pro	410	[26]
Met-Pro	870	[26]
Val-Pro	880	[26]
Pro-Gly-Pro-Ile-His-Asp-Ser	1000	[31]
Ile-Pro-Pro-Leu-The-Gln-Thr-Pro-Val	1300	[31]
Arg-Pro	2240	[26]

IC_50_: half-maximal inhibitory concentration

In one study, a zein protein hydrolysate with papain (ZeinH) was found to strongly stimulate GLP-1 secretion in the ileum rather than the duodenum or the jejunum in anesthetized rats [47]. This study also indicated that direct and indirect regulations of GLP-1 secretion mediate not only fat-induced GLP-1 secretion but also dietary peptide-induced GLP-1 secretion in the intestine. Further research was done to evaluate the antidiabetic effect of ZeinH in rats [48]. The ileal administration of ZeinH (500 mg) significantly decreased the level of glucose in plasma , increased insulin and active GLP-1 concentrations by up to 6.3- and 3.1- folds, respectively, as well as reduced DPP-IV activity by 26.8%, as compared to the control rats (deionized water). In addition, the oral administration of ZeinH (2 or 4 g/kg) showed significantly lower glucose levels in a dose-dependent manner after the glucose injection. The elevation of glucose concentration at 15 min in 4 g/kg ZeinH-treated rats was about half of the concentration that was found in control rats.

**Table 2:** — Summary of the research on the *in vivo* antidiabetic effect of peptides derived from food proteins.

Protein source	Sequence /IC50	Experimental models	Function	Reference
Gouda-type cheese	LPQNIPPL /46 μM	Female SD rats	Lower glucose levels in plasma	[31]
Zein		Male SD rats	Induce GLP-1 secretion and inhibit DPP-IV activity	[48]
Zein		Male SD rats	Stimulate GLP-1 secretion	[47]
Whey proteins		Healthy males	Increase insulin secretion	[49]
ß-lactoglobulin	VAGTWY/174 _	C57BL/6 mice	Decrease blood glucose level	[40]
Lysozyme	--/0.9 mg/mL	ZDF rats	Inhibit 25% of DPP-IV activity	[33]
Porcine skin gelatin	< 1 kDa	STZ-induced diabetic rats	Increase GLP-1 and insulin levels, inhibit DPP-IV activity, and decrease glucagon levels	[32]

The previous research mostly studied the acute treatment of peptides and their effects on some parts of hypoglycemic activity in normal animal models. Ergo, using diabetic animal models to investigate the antidiabetic effects of peptides may be able to clarify the peptides' real mechanisms and efficiency. The ZDF (Zucker Diabetic Fatty) rat model of T2DM has been used to evaluate the *in vivo* bioactivity of lysozyme/alcalase hydrolysate in inhibiting DPP-IV [33]. In acute treatment experiments, the hydrolysate and vildagliptin (positive control) were administered by oral gavage of a single dose and were evaluated over a 6 h period. The hydrolysate exerted significant inhibition, approximately 25% inhibition of plasma DPP-IV after 90 min, with a time pattern comparable to that observed after vildagliptin. However, the results of the changes in the associated modulation of metabolic products (glucose, insulin and GLP-1) were not reported. A previous study demonstrated that the peptides (PGH) in the < 1 kDa ultrafiltration fraction of the porcine skin gelatin hydrolysate showed great DPP-IV inhibitory activity and were used for an *in vivo* animal experiment [32]. A long-term (42 days) *in vivo* test of streptozotocin (STZ)-induced diabetic rats was used as the animal model to evaluate the anti-diabetic effects of the hydrolysates. Daily administration of PGH (300 mg/day) or sitagliptin (30 mg/day) was able to improve the glucose tolerance in the diabetic rats at days 21 and 42. The DPP-IV activities of the diabetic rats administered PGH and sitagliptin after 42 days were 50.0% and 31.0% lesser than the diabetic control rats, respectively. Furthermore, the PGH and sitagliptin treated rats had an increase of about 10% in active GLP-1 levels and 6-8 fold increase insulin levels as compared to the diabetic control rats. Therefore, the conclusion is that PGH had a superior antidiabetic effect in STZ-induced diabetic rats, including an improvement of glucose tolerance, an elevation of plasma insulin and GLP-1 levels, an inhibition of DPP-IV activity, and a reduction of gluca- gon levels.

Although there have been a lot of *in vitro* DPP-IV inhibitory peptides or protein hydrolysates reported, their *in vivo* effects on diabetic animals or patients have rarely been studied. More detailed *in vivo* studies to evaluate the efficacy, safety, bioavailability, and potency of inhibitory peptides and/or protein hydrolysates are needed.

## 4. In silico approaches to predict the potential of peptides as DPP-IV inhibitors

Food proteins are well known to be precursors of bioactive peptides, and these peptides can be released through *in vitro* hydrolysis by specific enzymes or fermentation by bacteria [50]. The traditional method to screen for the bioactive peptides from a protein involves selecting proteases that have the ability to truncate potent peptides according to the literature reports and *in vitro* experimental tests. However, the key roles of the protein sequence and the specificity of proteases in affecting the generation of bioactive peptides could make this approach costly and time- consuming [35]. Hence, several novel computational approaches to predict the potential of a protein to be the precursor of bioac- tive peptides by using the combinations of the protein sequences and enzyme specificity have been recently developed [35-38]. The availability of BIOPEP (http://www.uwm.edu.pl/biochemia/ index.php/en/biopep), a database of bioactive peptide sequences, allows for the theoretical prediction of potential bioactivity of different substrates and their corresponding activity after hydrolysis using enzymes with known cleavage specificities [51]. The most commonly used *in silico* method for... predicting the potential of a protein source as a DPP-IV inhibitor is the frequency of occurrence of bioactive fragments in protein sequences. Some researchers have predicted the presence of DPP-IV inhibitors in the sequences of various dietary proteins using the BIOPEP database [25, 37, 38, 52, 53]. They used already known information about the protein sequences and the DPP-IV inhibitory peptide sequences that are currently included in the UniProt Knowledgebase of ExPASy Proteomics Server (http://expasy.org) and BIO- PEP database. The potential of each selected protein is quantified based on the frequency of the occurrence of fragments matching peptides with DPP-IV inhibitory activity relative to the length of the protein chain using the following equation [54]:

A = a/ N

where A is the occurrence frequency, a is the number of peptides with DPP-IV activity within the protein chain, and N is the number of amino acid residues in the protein chain.

According to the results of a previous study [37], caseins from cow's milk and collagens from bovine meat and salmon were found to be the best precursors of DPP-IV inhibitors all with occurrence frequencies over 0.249. Although this kind of *in silico* approach does provide useful information on the potential of proteins to serve as bioactive peptides precursors, it does not allow the identification of the most potent proteins in terms of inhibitory activity. In consideration of the overlapping sequences and potency (IC50 value) of the DPP-IV inhibitory peptides embedded in protein sequences, two parameters have been established [38]: protein coverage (PC) value and potency index (PI). The corrected PC value only takes into account the most potent DPP-IV inhibitory peptide in overlapping areas of the protein sequence. The PI takes into account both the occurrence frequency and potency (IC_50_ value) of the peptides present within a given protein. This study has revealed that the bovine K-casein is the protein with the highest PI value of 17.89 μM^-1^g^-1^ [38], in particular the potent DPP-IV inhibitory peptide, Ile-Pro-Ile, was found in the sequence. This is in contrast to earlier results that showed that bovine ß-casein had the highest occurrence frequency (0.249) of DPP-IV inhibitory peptides, while bovine κ-casein had an occurrence frequency of only 0.130 [37].

Additionally, a docking analysis has also been used as an *in silico* approach to predict the affinity of the peptides to bind to the active site of DPP-IV. A previous study has shown that the peptide Trp-Trp-Trp had the best docking affinity, was a moderate DPP-IV inhibitor (IC_50_ 216 pM), and its action was noncompetitive [Nongonierma *et al.,* 2014]. The authors of the study suggested that the peptide may not bind to the active site of DPP- IV as assumed in the docking prediction. Their results revealed that there is no clear relationship between the docking affinity and the DPP-IV inhibitory activities of the peptides. In addition, they showed that the utilization of molecular docking can be a predictive tool for the competitive inhibitors. Thus, docking can be used as a preliminary tool to help focus experimental screening efforts on a smaller number of candidate peptides.

Since the studies relied entirely on the currently available data on DPP-IV inhibitory peptide sequences, it is possible that other fragments presenting even better DPP-IV inhibitory activity are presently unknown or have not yet been reported in the literature. Furthermore, the putative peptide sequences have to be released from their parent proteins to become active, and the final conclusions on the potential of dietary proteins for DPP-IV inhibitors can be drawn only after experimentally assessing the release of these bioactive peptides upon *in vitro* or *in vivo* hydrolysis. Further effort is therefore needed on the development of an *in silico* approach capable of being used as a screening tool for the evaluation of the potential of dietary proteins for the generation of *in vitro* and *in vivo* DPP-IV inhibitors.

## 5. Conclusions and future perspectives

The importance and scientific understanding of DPP-IV inhibitors that may improve glycemic control in T2DM patients has increased in the last few decades. Much work has been done with food protein-derived DPP-IV inhibitory peptides; however, the evidence of their *in vivo* antidiabetic effect needs to be built in more animal and clinical studies. The "BIOPEP" database is responsible for collecting all of the information about bioactive peptides from academic literature, and 707 protein sequences are also available. Further research into the establishment of *in silico* approaches to efficiently predict the potential of proteins as the precursors of DPP-IV inhibitory peptides is necessary.

The most challenging task in the DPP-IV inhibitory peptide research is the establishment of a detection model for identification of possible mechanisms by which they can exert *in vivo* antidiabetic activity. Albeit the simulated GI digestion is a kind of model mimicking the actions of human GI enzymes, the antidi- abetic effect of the peptides released from the DPP-IV inhibitory peptides through simulated GI digestion is not guaranteed to be similar to that through the GI tract *in vivo.* So the possible strategies for increasing the resistance to digestive enzymes as well as the cellular permeability of the peptides are two factors that also need to be investigated.
